# Prevalence and Global Initiative for Chronic Obstructive Lung Disease Group Distribution of Chronic Obstructive Pulmonary Disease Detected by Preoperative Pulmonary Function Test

**DOI:** 10.1371/journal.pone.0115787

**Published:** 2015-01-27

**Authors:** Sun Mi Choi, Jinwoo Lee, Young Sik Park, Chang-Hoon Lee, Sang-Min Lee, Jae-Joon Yim, Young Whan Kim, Sung Koo Han, Chul-Gyu Yoo

**Affiliations:** Division of Pulmonary and Critical Care Medicine, Department of Internal Medicine, Seoul National University College of Medicine, Seoul National University Hospital, Seoul, Republic of Korea; University of Athens Medical School, GREECE

## Abstract

**Background:**

Despite being a major public health problem, chronic obstructive pulmonary disease (COPD) remains underdiagnosed, and only 2.4% COPD patients are aware of their disease in Korea. The objective of this study was to estimate the prevalence of COPD detected by spirometry performed as a preoperative screening test and to determine the Global Initiative for Chronic Obstructive Lung Disease (GOLD) group distribution and self-awareness of COPD.

**Methods:**

We reviewed the medical records of adults (age, ≥40 years) who had undergone spirometry during preoperative screening between April and August 2013 at a tertiary hospital in Korea. COPD was defined as a postbronchodilator forced expiratory volume in 1 s/forced vital capacity ratio of <0.7. We analyzed self-administered COPD questionnaires for the assessment of the frequency of acute exacerbation over the previous year and dyspnea severity using the modified Medical Research Council dyspnea scale and COPD assessment test.

**Results:**

Among 3029 patients aged >40 years who had undergone spirometry as a preoperative screening test, 474 (15.6%; 404 men; median age, 70 years; range, 44–93 years) were diagnosed with COPD. Only 26 (5.5%) patients reported previous diagnosis of COPD (2.1%), emphysema (0.8%), or chronic bronchitis (2.5%). The GOLD group distribution was as follows: 63.3% in group A, 31.2% in group B, 1.7% in group C, and 3.8% in group D.

**Conclusions:**

The prevalence of COPD diagnosed by preoperative spirometry was 15.6%, and only 5.5% patients were aware of their disease. Approximately one-third of the COPD patients belonged to GOLD groups B, C, and D, which require regular treatment.

## Introduction

Chronic obstructive pulmonary disease (COPD) is characterized by persistent airflow limitation that is usually progressive and associated with an enhanced chronic inflammatory response to noxious particles or gases in the airways and lungs [1–3]. COPD is a major cause of morbidity and mortality, and its prevalence is increasing worldwide. It is associated with a high economic and social burden. Despite being a major public health problem, COPD remains underdiagnosed.

The prevalence of COPD varies among reports. A previous meta-analysis reported pooled prevalence estimates of 7.6% [4], and a recent report showed an estimated population prevalence [Global Initiative for Chronic Obstructive Lung Disease (GOLD) grade II and higher] of 10.1% [5]. COPD prevalence based on self-reporting is the lowest estimate. A recent meta-analysis showed that less than 6% of the adult population were aware of their COPD [4]. In Korea, only 2.4% COPD patients in a community population were aware of their disease [6].

Disease prevalence largely depends on the studied population. Several studies have reported the prevalence of COPD among patients with cardiovascular diseases [7], respiratory tract infections [2], and other conditions [8] that require medical management. The prevalence of COPD in these patients is 10.5–27%, which is higher than that reported in a field survey [4]. Because COPD, a systemic inflammatory disease, has been shown to be associated with many diseases that require medical management, the higher prevalence in such patients could be explained by a selection bias for COPD patients with comorbidities. For example, patients with conditions that require regular medical attention have more contact with physicians; therefore, they are more likely to be diagnosed with COPD.

Spirometry is mandatory for the diagnosis of COPD. A considerable number of patients is diagnosed with COPD by spirometry performed not for respiratory symptoms but for various other reasons. For example, although guidelines for preoperative screening do not recommend spirometry as a routine test [9], many surgeons perform spirometry for preoperative screening because COPD is one of the most important risk factors for postoperative pulmonary complications [10]. Nevertheless, there is limited data on the prevalence and severity of COPD among surgical candidates, and the numbers vary from 5% to 40% [3, 11, 12].

While diagnostic methods for COPD have remained unchanged, management strategies have significantly improved, particularly with regard to therapeutic intervention and disease assessment. Specifically, COPD assessment must consider all of the following four aspects: 1) patient’s symptoms, 2) severity of the airflow limitation measured by spirometry, 3) exacerbation risk, and 4) presence of comorbidities. As a result, the 2011 GOLD guideline suggested a new assessment method for COPD that considers the current level of symptoms and the exacerbation risk, with the ultimate goal to achieve better therapeutic outcomes [1, 13]. The guideline suggests that patients in groups B, C, and D should receive regular treatment [14].

The objective of this study was to estimate the prevalence of COPD among surgical candidates, determine the distribution of various COPD types based on the new classification defined in the 2011 GOLD guidelines, and assess patients’ self-awareness of COPD.

## Methods

We screened all patients who had undergone spirometry at a tertiary hospital in Korea, Seoul National University Hospital, between April 2013 and August 2013. We reviewed the departments that had administered spirometry tests and included patients referred from surgical departments, because spirometry was likely administered as part of preoperative screening. Finally, we reviewed medical records in order to identify patients who planned to undergo elective surgery.

We defined COPD as a postbronchodilator (post-BD) forced expiratory volume in 1 s (FEV_1_)/forced vital capacity (FVC) ratio of <0.7 in patients aged ≥40 years, according to the GOLD guideline [1]. Spirometry results were expressed as percentages of the predicted values and were calculated using Morris’s predictive equations as follows: predicted FEV_1_ for men = 0.092 × height − 0.032 × age − 1.26, predicted FEV_1_ for women = 0.089 × height − 0.025 × age − 1.932, predicted FVC for men = 0.148 × height − 0.025× age − 4.241, predicted FVC for women = 0.115 × height − 0.024× age − 2.852 (height in inches) [15].

Spirometry was performed using spirometers (Model: VMAX229, VMAX22, and VMAX 20; SensorMedics, Yorba Linda, CA) by five trained technicians according to the 2005 American Thoracic Society (ATS)/European Respiratory Society (ERS) recommendations [16].

Data collected included demographic characteristics; surgery-related factors such as type of surgery, surgical duration, and anesthesia type; and whether the patient began to visit a pulmonologist after surgery. We also analyzed two different self-administered questionnaires collected at the time of the pulmonary function test (PFT). One questionnaire focused on the past medical and smoking histories. Specifically, the patients were asked to identify whether they had previously been diagnosed with a respiratory disease, and, if yes, which specific disease, namely COPD, chronic bronchitis, emphysema, asthma, bronchiectasis, tuberculosis, pneumonia, and others. The other questionnaire was completed by COPD patients (postbronchodilator FEV_1_/FVC < 0.7) to evaluate the risk of acute exacerbation of COPD and dyspnea severity. Patients were asked to identify the frequency and severity of acute exacerbation in the preceding year. The resulting data were used to assess the exacerbation risk according to the GOLD guideline, i.e., one or more hospitalizations for COPD exacerbations or two or more COPD exacerbations indicated a high risk [14]. We also evaluated dyspnea severity using the modified Medical Research Council (mMRC) dyspnea scale and the COPD assessment test (CAT) score.

COPD patients who completed the second questionnaire were classified into the following four groups based on the results of spirometry and questionnaires: group A, low risk of exacerbations and low level of symptoms; group B, low risk of exacerbations and high level of symptoms; group C, high risk of exacerbations and low level of symptoms; and group D, high risk of exacerbations and high level of symptoms [14].

Statistical analyses were performed using SPSS 17.0 (Chicago, IL, USA). Variables were compared between patients with and without COPD using Student’s *t*-test or the Mann—Whitney *U* test for continuous variables and the χ^2^ test or Fisher’s exact test for categorical variables in univariate analyses.

### Ethics statement

This study was approved by the Seoul National University Institutional Review Board for Clinical Research (IRB number: 1308–084–514). The informed consent requirement was waived because of the retrospective study design. All patient records and information were anonymized and deidentified prior to analysis.

## Results

### Baseline characteristics

**Fig 1:**
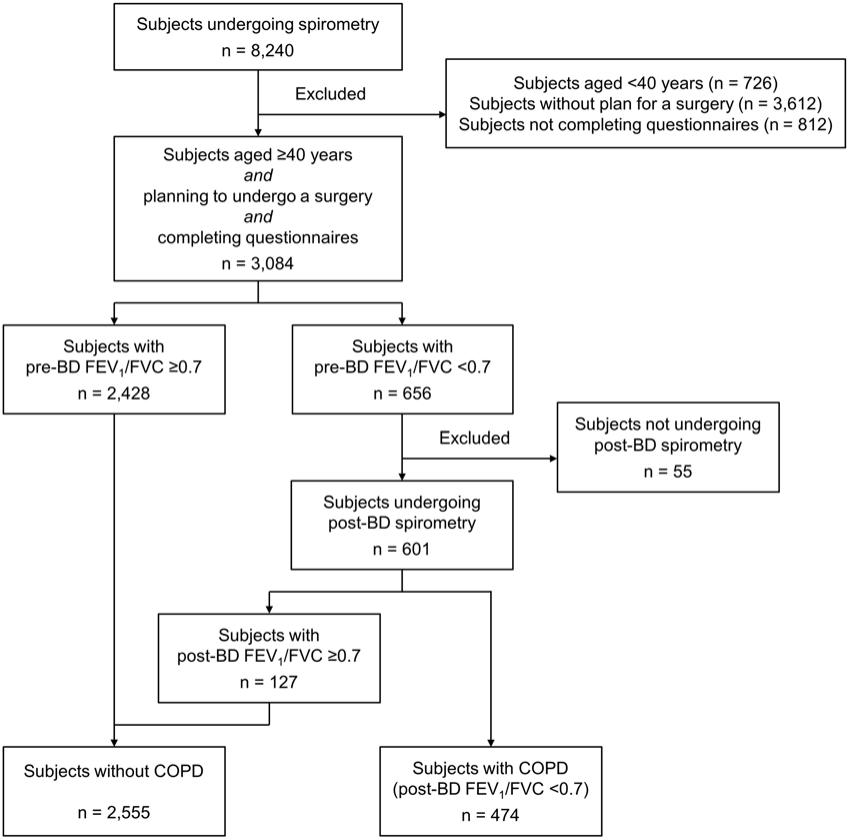
Selection of subjects for the analysis of COPD prevalence among surgical candidates. BD: bronchodilator, FEV1/FVC: forced expiratory volume in 1 s/forced vital capacity, COPD: chronic obstructive pulmonary disease.

A total of 8240 patients underwent PFT during the study period (see Fig. 1 for the patient selection process). After a review of medical records, we excluded 726 patients aged <40 years and 3618 patients who had undergone PFT for other medical conditions and not for preoperative screening. We also excluded 812 patients who did not complete the questionnaires. Among the remaining 3084 patients, 656 had a pre-BD FEV_1_/FVC of <0.7. We excluded 55 patients who did not undergo post-BD spirometry. Among the remaining 601 patients, 127 had a post-BD FEV_1_/FVC of ≥0.7. Eventually, 474 (15.6%) patients were found to have COPD with a post-BD FEV_1_/FVC of <0.7.

The median age of patients was 64 years, and 53.9% were men. Surgeries were performed in the following departments: General Surgery (41.5%), Thoracic and Cardiovascular Surgery (3.2%), Orthopedic Surgery (13.4%), Urology (19.1%), Neurosurgery (9.7%), Plastic and Reconstructive Surgery (3.6%), Otorhinolaryngology (5.8%), Obstetrics and Gynecology (5.9%), and Ophthalmology (0.5%) departments. Among the COPD patients, 85.2% were men, and the median age was 70 years. Table 1 shows the baseline characteristics of the COPD and non-COPD patients. COPD patients were older than non-COPD patients (p < 0.001) and were predominantly men (p < 0.001). The number of patients who underwent surgeries under general anesthesia was higher in the non-COPD group than in the COPD group (p = 0.011), while a higher number of patients in the COPD group underwent surgeries under spinal anesthesia (p = 0.001). The number of patients referred to a pulmonologist for preoperative evaluation was higher in the COPD group than in the non-COPD group (p < 0.001).

**Table 1:** Baseline characteristics.

	**Non-COPD** **N = 2555 (84.4)**	**COPD** **N = 474 (15.6)**	**p-value**
Age (range)	63 (40–92)	70 (44–93)	<0.001
Men	1217 (47.6)	404 (85.2)	<0.001
BMI, Kg/m^2^	23.8 (21.7–26.0)	23.6 (21.9–25.5)	0.245
Smoking			<0.001
Current smoker	328 (12.9)	97 (20.7)	
Ex-smoker	774 (30.5)	264 (56.4)	
Never smoker	1433 (56.5)	107 (22.9)	
Baseline ABGA*			
pH	7.39 (7.36–7.42)	7.40 (7.38–7.42)	0.013
PaO_2_, mmHg	102.0 (88.5–133.3)	91.8 (82.9–107.8)	<0.001
PaCO_2_, mmHg	40.6 (37.7–43.7)	40.2 (37.2–43.1)	0.164
HCO_3_, mmol/L	24 (22.6–25.5)	24.4 (23.1–25.3)	0.102
Spirometry^†^			
FEV_1_, L	2.5 (2.1–3.0)	2.2 (1.7–2.5)	<0.001
FEV_1_, %	111 (100–121)	89 (76–102)	<0.001
FVC, L	3.2 (2.7–3.9)	3.6 (3.0–4.1)	<0.001
FVC, %	102 (93–112)	101 (90–113)	0.110
FEV_1_/FVC, %	77 (74–81)	62 (56–65)	<0.001
Type of anesthesia^‡^			
General anesthesia	1948 (87.6)	334 (82.7)	0.011
Spinal anesthesia	186 (8.4)	55 (13.6)	0.001
Others	90 (4.0)	15 (3.7)	0.890
Surgical duration, min	150 (90–231)	140 (79–245)	0.279
Consultation with pulmonologists before surgery	494 (19.3)	313 (66.0)	<0.001

Data are presented as numbers (%) or medians (interquartile range) unless otherwise indicated.

COPD: chronic obstructive pulmonary disease, BMI: body mass index, ABGA: arterial blood gas analysis, PaO_2_: partial pressure of oxygen in arterial blood, PaCO_2_: partial pressure of carbon dioxide in arterial blood, FEV_1_: forced expiratory volume in 1 s, FVC: forced vital capacity

*missing: 1839,

^†^pre-bronchodilator,

^‡^missing: 494.

Although preoperative arterial blood gas analysis (ABGA) was not performed for all patients, it showed a lower partial pressure of oxygen in arterial blood (PaO_2_), which was still within the normal range (74–108 mmHg), in COPD patients (p < 0.001). On the other hand, the partial pressure of carbon dioxide in arterial blood (PaCO_2_) did not differ between the two groups (Table 1). The number of patients with a history of smoking was higher in the COPD group than in the non-COPD group (p < 0.001).

### Awareness of COPD

Among the 474 COPD patients, 61.2% reported undergoing spirometry at least once. However, only 26 (5.5%) patients reported a previous diagnosis of COPD (2.1%), emphysema (0.8%), or chronic bronchitis (2.5%) by a physician (Table 2). On the other hand, 54 (11.3%) patients declared that they had been diagnosed with asthma. Among them, 20 showed a positive bronchodilator response, defined as a ≥12% increase and 200 mL of pre-BD FEV_1_ after inhalation of the short-acting bronchodilator salbutamol. Sixty-one (12.9%) of the 474 patients reported the use of an inhaler for their respiratory disease. Only nine patients among the 31 who had been followed by a pulmonologist in the same hospital were aware of their COPD; 11.1% COPD patients began visiting a pulmonologist after their surgeries.

**Table 2:** Medical histories reported in the self-administered questionnaire.

		**Non-COPD** **N = 2555 (84.4)**	**COPD** **N = 474 (15.6)**	**p-value**
Previous spirometry testing		1350 (53.4)	290 (61.2)	0.002
Regular visit to a clinic		1356 (53.1)	272 (57.4)	0.090
Regular health screening		2224 (87.5)	413 (87.1)	0.932
Respiratory disease				
COPD	COPD*	9 (0.4)	10 (2.1)	<0.001
	Emphysema*	6 (0.2)	4 (0.8)	0.057
	Chronic bronchitis	28 (1.1)	12 (2.5)	0.012
Asthma		93 (3.6)	54 (11.4)	<0.001
Bronchiectasis*		21 (0.8)	12 (2.5)	0.001
Tuberculosis		172 (6.7)	53 (11.2)	0.001
History of pneumonia		49 (1.9)	8 (1.7)	0.788
Lung cancer*		20 (0.8)	8 (1.7)	0.068
Hypertension		429 (16.8)	82 (17.4)	0.703
Diabetes mellitus		229 (9.0)	42 (8.9)	0.941
Hypercholesterolemia		80 (3.1)	11 (2.3)	0.342
Liver disease		57 (2.2)	8 (1.7)	0.453
Renal disease		29 (1.1)	9 (1.9)	0.170
Cardiovascular disease		130 (5.1)	33 (7.0)	0.097
Cerebrovascular disease		52 (2.0)	12 (2.5)	0.491
Musculoskeletal disease		120 (4.7)	20 (4.2)	0.648
Thyroid disease		79 (3.1)	4 (0.8)	0.006
Malignant disease		206 (7.6)	47 (10.0)	0.131

Data are presented as numbers (%).

COPD: chronic obstructive pulmonary disease.

*Fisher’s exact test.

### Past medical history

Table 2 shows the results of self-administered questionnaires on past medical histories. The number of patients with a history of asthma (p < 0.001), bronchiectasis (p = 0.001), and tuberculosis (p = 0.001) was higher in the COPD group than in the non-COPD group. The rate of other comorbidities, including hypertension, diabetes mellitus, liver disease, renal disease, cardiovascular disease, cerebrovascular disease, and malignant disease, was similar in both groups. Thyroid disease was more common in the non-COPD group (p = 0.006), probably because there were more females in this group. The number of patients who underwent regular health screenings was similar in both groups (p = 0.932).

### Results of spirometry

The majority of COPD patients (78.5%) had mild airflow limitation with a post-BD FEV_1_ of ≥0.8, followed by moderate (18.4%), severe (3.05), and very severe (0.2%) airflow limitation (Table 3). Sixty-eight (14.3%) COPD patients showed a positive bronchodilator response.

**Table 3:** Distribution of airflow limitation, level of symptoms, and exacerbation of COPD among COPD patients.

**Post-BD FEV_1_** **(% predicted)**	**mMRC≥ 2**	**CAT ≥ 10**	**Experience of** **exacerbation**	**Severe** **exacerbation**	**Total**
FEV_1_ ≥ 80	44 (11.8)	92 (24.7)	7 (1.9)	5 (1.3)	372 (100)
50 ≤ FEV_1_ < 80	29 (33.3)	37 (42.5)	9 (10.3)	2 (2.3)	87 (100)
30 ≤ FEV_1_ < 50	7 (50)	7 (50)	2 (14.3)	2 (14.3)	14 (100)
FEV_1_ < 30	0 (0)	0 (0)	0 (0)	0 (0)	1 (100)
Total	80 (16.9)	136 (28.7)	18 (3.8)	9 (1.9)	474 (100)

Data are presented as numbers (%).

Post-BD: postbronchodilator, FEV1: forced expiratory volume in 1 s, mMRC: modified Medical Research Council, CAT: COPD assessment test.

### Assessment of risk of exacerbation and level of symptoms

All 474 COPD patients completed the second questionnaire assessing the risk of acute exacerbation of COPD and dyspnea severity. Of these, 18 (3.8%) reported at least one exacerbation in the preceding year. Nine (1.9%) patients were identified to have a high risk of exacerbation, as explained in the Methods section (Table 3). The median mMRC dyspnea and CAT scores were 1 (interquartile range [IQR], 0–1) and 6 (IQR, 3–10), respectively.

The GOLD group distribution of COPD patients based on the mMRC score was 79.7% (378) in group A, 14.8% (70) in group B, 3.4% (16) in group C, and 2.1% (10) in group D. The distribution was significantly different (p < 0.001) when the CAT score was used, i.e., 69.0% (327) in group A, 25.5% (121) in group B, 2.3% (11) in group C, and 3.2% (15) in group D (Table 4). The GOLD group distribution using either the mMRC or CAT score, whichever indicated a higher level of symptoms, was as follows: 63.3% in group A, 31.2% in group B, 1.7% in group C, and 3.8% in group D.

**Table 4:** Distribution of patients* into GOLD categories according to symptom assessment (mMRC versus CAT scores).

	**mMRC scores**		**CAT scores**	
	**mMRC 0–1**	**mMRC ≥ 2**	**CAT < 10**	**CAT ≥ 10**
Symptom category				
A	378 (79.7)		327 (69.0)	
B		70 (14.8)		121 (25.5)
C	16 (3.4)		11 (2.3)	
D		10 (2.1)		15 (3.2)
Symptom subcategories				
C1^†^	8 (1.7)		8 (1.7)	
C2^‡^	8 (1.7)		3 (0.6)	
C3^§^	0		0	
D1^†^		5 (1.1)		5 (1.1)
D2^‡^		3 (0.6)		8 (1.7)
D3^§^		2 (0.4)		2 (0.4)

*N = 474

^†^Meets FEV1 criteria (<50% FEV1 predicted).

^‡^Meets exacerbation criteria (≥2 COPD exacerbations or ≥1 hospitalization or visit to the emergency department because of COPD exacerbation in past 12 months).

^§^Meets exacerbation and FEV1 criteria (<50% FEV1 predicted and ≥2 COPD exacerbations or ≥1 hospitalization or visit to the emergency department because of COPD exacerbation in the past 12 months).

## Discussion

In the current study, 474 patients (15.6%) were identified to have COPD among surgical candidates in a tertiary hospital, with the GOLD group distribution as follows: 63.3% in group A, 31.2% in group B, 1.7% in group C, and 3.8% in group D. Only 26 (5.5%) patients reported awareness of their disease.

The COPD prevalence rate of 15.6% among the surgical candidates in this study was similar to that in a community population in Korea. Two previous studies reported on the prevalence of COPD in Korea, which was 13.4% among patients aged >40 years and 17.2% among those aged >45 years according to population-based spirometry surveys performed in 2008 and 2001, respectively [6, 17].

A recent study reported a COPD prevalence rate of 4.82% among surgical patients [18], which was lower than that in this study. However, that study defined COPD using more stringent criteria, such as a predicted FEV_1_ of <75% or hospitalization. On the other hand, we applied the criteria established by the GOLD guideline and included the milder form of disease, which resulted in the higher prevalence.

Patients with COPD in our study were older (p < 0.001) and predominantly male (p < 0.001), consistent with the well-known characteristics of COPD patients. Weight loss is often observed in COPD patients [18, 19]; however, the body mass index (BMI) did not differ between patients with and without COPD in the present study. Similarly, several other studies have reported similar or even higher BMI values for COPD patients than for healthy controls [20, 21].

Smoking is the most important risk factor for COPD. The number of patients with a smoking history was significantly higher among COPD patients than among non-COPD patients in this study (p < 0.001). In total, 23% COPD patients were never smokers, similar to the findings in previous reports on large populations in the US, UK, and Spain [22–24].

When assessing surgery-related parameters, we found that the surgical duration was similar between the two groups. However, a significantly higher number of patients with COPD had undergone surgeries under spinal anesthesia, while a higher number of patients without COPD had undergone surgeries under general anesthesia. Old age and COPD are important risk factors for postoperative pulmonary complications, particularly in patients undergoing surgery under general anesthesia. Therefore, surgeons are likely to opt less frequently for surgery under general anesthesia in COPD patients. This notion is further supported by the fact that a higher number of patients with COPD was referred to pulmonologists prior to surgery (p < 0.001).

Compared with non-COPD patients, a significantly higher number of COPD patients reported respiratory diseases, including asthma, bronchiectasis, and tuberculosis (Table 2). Various respiratory diseases, including asthma and tuberculosis, could be risk factors for COPD [25, 26], and vice versa. In addition, a previous study addressing the overlap between various obstructive airway diseases showed that 17–19% patients had more than one condition, with the overlap increasing with advancing age [27].

Among several conditions, asthma–COPD overlap syndrome (ACOS) was the most common in older patients [28]. ACOS is characterized by persistent airflow limitation with clinical features associated with asthma. In the current study, 54 (11.3%) COPD patients reported a previous diagnosis of asthma, and an additional 48 (10.1%) patients showed a positive bronchodilator response. Thus, 21.4% COPD patients may be considered to have ACOS in the present study.

The severity of airflow limitation is considered a very important factor related to the prognosis of COPD. Similarly, symptoms such as dyspnea are recognized as important predictors of future mortality risk [29]. In addition, COPD exacerbations not only induce a decline in lung function but also increase the risk of death. However, the correlation between FEV_1_, symptoms and the health-related quality of life was relatively weak (r = −0.23, p < 0.0001) [30]. Based on these findings, the 2011 GOLD guideline proposed a new classification system for COPD according to symptoms, risk of exacerbation, and airflow limitation.

In our study, 78.4%, 18.4%, 3.9%, and 0.2% COPD patients had mild, moderate, severe, and very severe airflow limitation, respectively, according to the GOLD guideline (Table 3). Table 3 also shows the proportion of patients with a high level of symptoms when the mMRC and CAT scores were used for each grade of airflow limitation. For the severe and very severe grades of COPD, the mMRC and CAT scores identified a similar number of patients with a high level of symptoms, while the CAT score identified a higher number of such patients in the mild and moderate COPD groups than that identified by the mMRC score.

The 2011 GOLD guideline recommends the selection of one measure, either the mMRC or CAT score, to assess symptom severity. The mMRC score is easy to apply in practice; however, it only evaluates dyspnea severity. On the other hand, the CAT score may be used to evaluate the effects of COPD on the patients’ quality of life, and it is a multidimensional tool that assesses not only dyspnea but also other respiratory symptoms, general condition, and performance status.

A previous study reported a similar distribution of GOLD groups when the mMRC and St George’s Respiratory Questionnaire (SGRQ) were used [31]. In the current study, however, the distribution of GOLD groups differed depending on the method of symptom assessment, i.e., the mMRC or CAT score (Table 4). More patients were identified to belong to group B with the CAT score than with the mMRC (p < 0.001). Although the number of patients in groups C and D was relatively smaller than that in groups A and B, we further stratified patients in groups C and D as suggested in the previous study; C1 and D1 showed an FEV_1_ of <50% only, C2 and D2 met the exacerbation criteria only, and C3 and D3 met both the exacerbation and FEV_1_ criteria [31]. The number of patients in groups C1 and D1 was not higher than that in groups C2–3 and D2–3, while most patients classified into groups C and D in the previous study were classified into C1 and D1 groups, respectively. One possible explanation for this difference is the fact that the study excluded patients with a history of recent exacerbation of COPD treated with antibiotics or steroids, contributing fewer patients to the C2–3 and D2–3 groups than to the C1 and D1 groups [32].

When we used both measures and chose the higher score between mMRC and CAT, the GOLD group distribution was as follows: 63.3% in group A, 31.2% in group B, 1.7% in group C, and 3.8% in group D. According to this classification, 6.1% group B patients and 16.7% group D patients were aware of their COPD, while 3.7% group A patients and 37.5% group C patients were aware of their condition (p = 0.221). Considering the fact that patients in groups B and D had a high level of symptoms, the awareness of COPD was not related to the level of symptoms.

Several studies have described the underdiagnosis and under-recognition of COPD [24, 33, 34]. Recently, a study reported that only 2.4% COPD patients in Korea were aware of their condition [6]. In the present study, 5.5% COPD patients were aware of their COPD. In addition, even among patients who were followed up by a pulmonologist in the same medical center where this study was performed, only 30% reported a previous diagnosis of COPD, emphysema, or chronic bronchitis. One possible explanation is that the majority of these patients only had mild airflow limitation that did not require treatment.

The present study has several limitations. First, it was a retrospective, single-center analysis, and some selection bias may have occurred. For example, we excluded patients who had not completed the questionnaires, which might have introduced a selection bias. However, the retrospective design most likely did not affect the outcomes, because the objective was to assess the prevalence and group distribution rather than the efficacy of an intervention. Second, we used a fixed FEV_1_/FVC ratio to define airflow limitation, which may have resulted in a false positive diagnosis of COPD in our study, particularly in elderly subjects, rather than using a cutoff level based on the lower limit of normal values for FEV_1_/FVC. Third, the number of patients in groups B, C, and D was too small to draw general conclusions. Fourth, some data for surgery-related parameters were missing, because some patients had not yet undergone surgery. Fifth, we included patients with overlap syndromes that would usually be excluded from clinical trials for COPD. Sixth, the medical histories were evaluated primarily through self-reported questionnaires. With regard to the diagnosis of asthma, information on the history of allergic disease or atopy, IgE levels, results of the skin prick test, or fractional exhaled nitric oxide levels was not available because of the retrospective study design.

Despite the abovementioned limitations, to our knowledge, this is the first study to describe the prevalence of COPD using a GOLD group distribution analysis for surgical candidates. We also evaluated patient self-awareness of COPD and the ACOS.

In conclusion, the prevalence of COPD among surgical candidates aged ≥40 years was 15.6% in this study, with 78.5% patients having mild airflow limitation according to the GOLD grade. Approximately one-third of the COPD patients belonged to GOLD groups B, C, and D, requiring regular treatment. Only a small number of patients with COPD (5.5%) were aware of their condition, and COPD awareness was not related to symptom severity. Further research is required to identify the reasons for the low awareness of COPD and to thereby develop approaches for better management of the disease.
